# Assessment of the Macular Microvasculature in High Myopes With Swept Source Optical Coherence Tomographic Angiography

**DOI:** 10.3389/fmed.2021.619767

**Published:** 2021-05-17

**Authors:** Chee-Wai Wong, Saiko Matsumura, Hla Myint Htoon, Shoun Tan, Colin S. Tan, Marcus Ang, Yee-Ling Wong, Rupesh Agrawal, Charumati Sabanayagam, Seang-Mei Saw

**Affiliations:** ^1^Singapore National Eye Centre, Singapore Eye Research Institute, Singapore, Singapore; ^2^Duke-NUS Medical School, Singapore, Singapore; ^3^National Healthcare Group Eye Institute, Tan Tock Seng Hospital, Singapore, Singapore; ^4^Saw Swee Hock School of Public Health, National University of Singapore, Singapore, Singapore; ^5^R&D Vision Sciences Asia, Middle East, Russia and Africa (AMERA), Essilor International, Singapore, Singapore

**Keywords:** high myopia, swept source OCT angiography, macular microvasculature, foveal avascular zone, macular vessel density

## Abstract

**Background:** The risk of pathologic myopia (PM) increases with worsening myopia and may be related to retinal microvasculature alterations. To evaluate this, we analyzed the macular microvasculature of myopes with swept source-optical coherence tomographic angiography (SS-OCTA) in adolescent and young adult Singaporeans.

**Methods:** This is a prevalent case-control study including 93 young Chinese from the Strabismus, Amblyopia and Refractive error in Singaporean children (STARS, *N* = 45) study and the Singapore Cohort Study of Risk Factors for Myopia (SCORM, *N* = 48) studies. Macular vessel density (VD) measurements were obtained from 3 × 3 mm SS-OCTA scans and independently assessed using ImageJ. These measurements were compared between individuals with non-high myopia [non-HM, *N* = 40; SE >-5.0 diopter (D)] and HM (SE ≤-5.0D, *N* = 53).

**Results:** The mean macular VD was 40.9 ± 0.6% and 38.2 ± 0.5% in the non-HM and HM, groups, respectively (*p* = 0.01 adjusted for age and gender). Mean FAZ area in the superficial layer was 0.22 ± 0.02 mm^2^ in the HM group, which was smaller compared to non-HM group (0.32 ± 0.03 mm^2^, *p* = 0.04). Mean deep FAZ area was similar between the two groups (0.45 ± 0.03 mm^2^ and 0.48 ± 0.04 mm^2^ in the HM and non-HM groups, respectively, *p* = 0.70).

**Conclusions:** VD was lower and superficial FAZ area was smaller, in adolescents and young adults with HM compared to non-HM. These findings require validation in prospective studies to assess their impact on the subsequent development of PM.

## Background

Pathologic myopia (PM) is a sight threatening condition seen in highly myopic eyes characterized by posterior staphyloma, chorioretinal atrophy, tractional maculopathy and choroidal neovascularization. It is a major cause of visual impairment and blindness in Asia and worldwide (1–3). The risk of PM increases with increasing degree of myopia (4, 5), but whether this increased risk is due to axial elongation or to alterations of the retinochoroidal vasculature or both is still unclear. Emerging evidence suggests the latter (6–11). Up till recently, methods of studying the vasculature have been either not sensitive enough to study the macular microvasculature (Color Doppler ultrasonography) (12, 13) or too invasive (indocyanine green angiography). Optical coherence tomographic angiography (OCTA) allows non-invasive and depth resolved imaging of the superficial and deep retinal vasculature (14, 15). These novel clinical tools may potentially help to accurately characterize the retinal vasculature in young high myopes and predict their risk of visual impairment in the future.

Several studies have studied retinal vascular changes in high myopes compared to emmetropes or non-high myopes (14–21). The vascular parameters studied include vascular branching (fractal dimensions), vessel density (VD), foveal avascular zone (FAZ) area and retinal blood flow. Most, but not all of these studies have found decreased retinal VD and vascular branching in high myopes. All of these studies were conducted using spectral domain OCTA (SD-OCTA) and were performed in hospital-based cohorts. Swept source OCTA (SS-OCTA) has higher depth penetration and lower signal drop off compared to spectral domain, and has been shown to offer some advantages in the imaging of high myopes with long axial length (AL) or deep posterior staphyloma (3). Measurement of retinal vascular parameters in high myopes using SS-OCTA may thus offer a different perspective compared to SD-OCTA. In addition, hospital-based cohorts are inherently different from population-based cohorts, in particular with respect to selection bias where cases selected into a hospital setting may have more severe disease than similar cases from the community, or where controls from a hospital setting may have other co-existing conditions which are not present in participants from the general population.

To address these gaps, we conducted this study to evaluate the macular microvasculature with SS-OCTA in adolescent and young adult Singaporeans.

## Methods

We conducted a prevalent case-control study including participants from the last follow-up visit of two independent population-based studies: The Strabismus, Amblyopia and Refractive error in Singaporean children (STARS) and the Singapore Cohort Study of Risk Factors for Myopia (SCORM) studies. We included 93 children for the current study (*N* = 45 children aged 9–14 in 2017 from STARS and *N* = 48 aged 9–14 years in 2016 from SCORM). Only Chinese participants were included and none underwent refractive surgery.

The STARS study is a population-based survey of Chinese children aged 6–72 months residing in the government apartments in Singapore (22). Overall prevalence of myopia [Spherical Equivalent (SE) ≤-0.50 diopter (D)] and high myopia (HM) (SE ≤-6.00D) at baseline were 11.0% and 0.2%, respectively (23). Forty-seven children aged 9–14 years participated in a pilot 10-year recall study in 2017. A total of 45 subjects were included after excluding subjects without OCTA imaging (*N* = 2). The mean SE was −2.80D ± 1.72 (range, +1.55D to −6.10D). The proportion of myopia was 77.8% (*N* = 35). The proportion of HM and non-HM was 11.1% (*N* = 5) and 88.9% (*N* = 40), respectively.

The SCORM study, established in 1999, is the first myopia cohort study in Asia. Children aged 7–9 years were recruited and prior publications described the methodology of the SCORM study in detail (24). Fifty-two young adults with HM (SE ≤-5.00D) aged 22–26 years were followed up in 2016. A total of 48 Chinese subjects were considered eligible after excluding non-Chinese subjects (*N* = 3) and subjects without OCTA imaging (*N* = 1). All included subjects had HM. The mean SE was −7.43D ± 1.65 (range, −5.00D to −11.38D) in the SCORM study. Written informed consent was obtained from all participants in both the STARS and SCORM studies before each examination. The tenets of the Declaration of Helsinki were observed, and the study was reviewed and approved by the Ethics Committee of the Singapore Eye Research Institute.

### Eye Measurements

Cycloplegic autorefraction was performed by trained eye professionals. Cycloplegia was induced with three drops of 1% cyclopentolate 5 min apart in the STARS study, and with two drops of 1% tropicamide 5 min apart in the SCORM study. At least 30 min after the last drop, five consecutive refraction and keratometry readings were measured using an autokeratorefractometer (model RK5; Canon, Inc., Ltd., Tochigiken, Japan).

### Foveal Avascular Zone and Vessel Density Measurements

Swept-source optical coherence tomography (SS-OCT; DRI OCT Triton, Topcon, Japan) was obtained after pupil dilation. Both studies followed the same imaging protocol. The swept-source OCT-A images were all processed using the angiography ratio analysis (ARA) method (25). Volumetric OCT scans were acquired over a 3 mm × 3 mm field of view and each B-scan position was repeatedly scanned four times. Segmentation for the superficial vessel plexus (SVP) was performed with an inner boundary set at 3 μm beneath the internal limiting membrane (ILM) and the outer boundary was set at 15 μm beneath the inner plexiform layer (IPL). The deep vessel plexus (DVP) was segmented with an inner boundary at 15 μm beneath the IPL and the outer boundary at 70 μm beneath the IPL.

**Figure 1:**
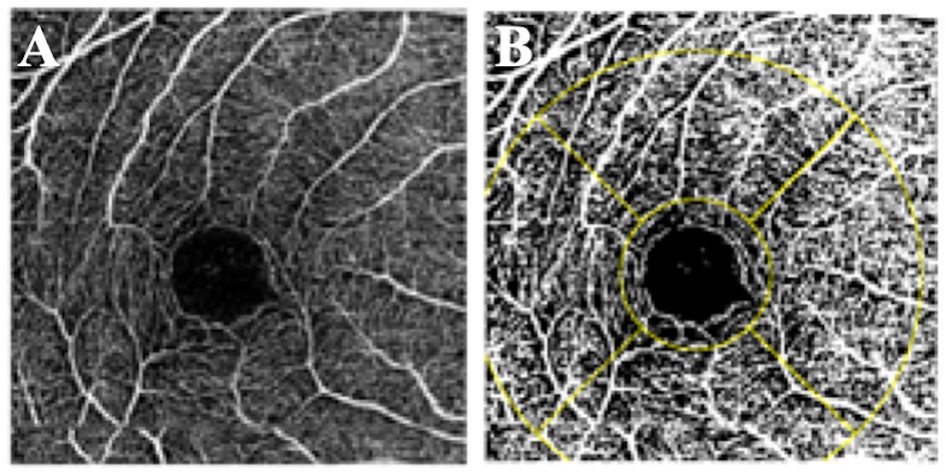
Vessel density measurement. **(A)** OCT-A scan of the superficial retinal plexus. **(B)** Binarized image with the ETDRS grid overlaid over the fovea.

OCT-A scans of the superficial and deep retinal vasculature were exported and independently assessed by two trained graders using ImageJ (version 1.49, National Institutes of Health, Bethesda, MD, USA). Graders were masked to patient details. After the scale was set to 320 pixels/3 mm, superficial and deep FAZ boundaries were manually traced by the graders as previously described (26), and the FAZ area were automatically calculated by the software. To determine VD, the image was converted to 8 bit and binarized using a pre-selected auto-thresholding method, Li Global Thresholding (Figure 1). Exported images from the device were first converted to 8-bit images (i.e., with 256 possible values per pixel). These were then automatically thresholded with the Li thresholding algorithm within ImageJ. The Li algorithm is an iterative technique which calculates a threshold *via* minimizing the local cross-entropy values (27). No manual input is required for this calculation. A grid comprising of the region of interest (ROI) was then demarcated around the central 1 and 3 mm Early Treatment Diabetic Retinopathy Study (ETDRS) subfields. The 3-mm zone was divided into superior, inferior, temporal, and nasal subfields. The vessel densities were obtained from each of these subfields. VD of each segment was defined as the arithmetic percentage of area occupied by retinal vessels in the thresholded image. This was automatically calculated and no additional manual input was required.

### Statistical Analysis

Our main outcome measures were mean overall macular VD and mean FAZ area. SE was defined as sphere plus half negative cylinder. The participants were categorized into two groups: non-HM (SE >-5.0 D) and HM (SE ≤-5.0D). The right eye of each participant was analyzed. The differences in means were evaluated using independent *t*-tests. ANCOVA was performed, adjusted for age and gender, for the comparisons of FAZ area and VD between groups. Correlation between SE and microvascular parameters were analyzed with Pearson's correlation coefficient. Adjustments for multiple comparisons was made with Bonferroni correction. Statistical significance was set at *P* = 0.05. All statistical analyses were performed with SPSS (IBM Corp. IBM SPSS Statistics for Windows, Version 24.0. Armonk, NY: IBM Corp.).

## Results

### Baseline Characteristics

A total of 93 participants (45 from the STARS study and 48 from the SCORM study) were included in the analysis. The participants were categorized into two groups: non-HM (SE >-5.0D, *N* = 40) and HM (SE ≤ -5.0D, *N* = 53). The mean age was 22.9 ± 3.7 years (female, 43.4%) in HM group and 10.9 ± 1.6 years (female, 52.5%) in non-HM group (*p* < 0.001). The mean SE was −7.28D ± 1.64 and −2.42D ± 1.42 in HM group and non-HM group, respectively (*p* < 0.001).

### Foveal Avascular Zone and Vessel Density

**Figure 2:**
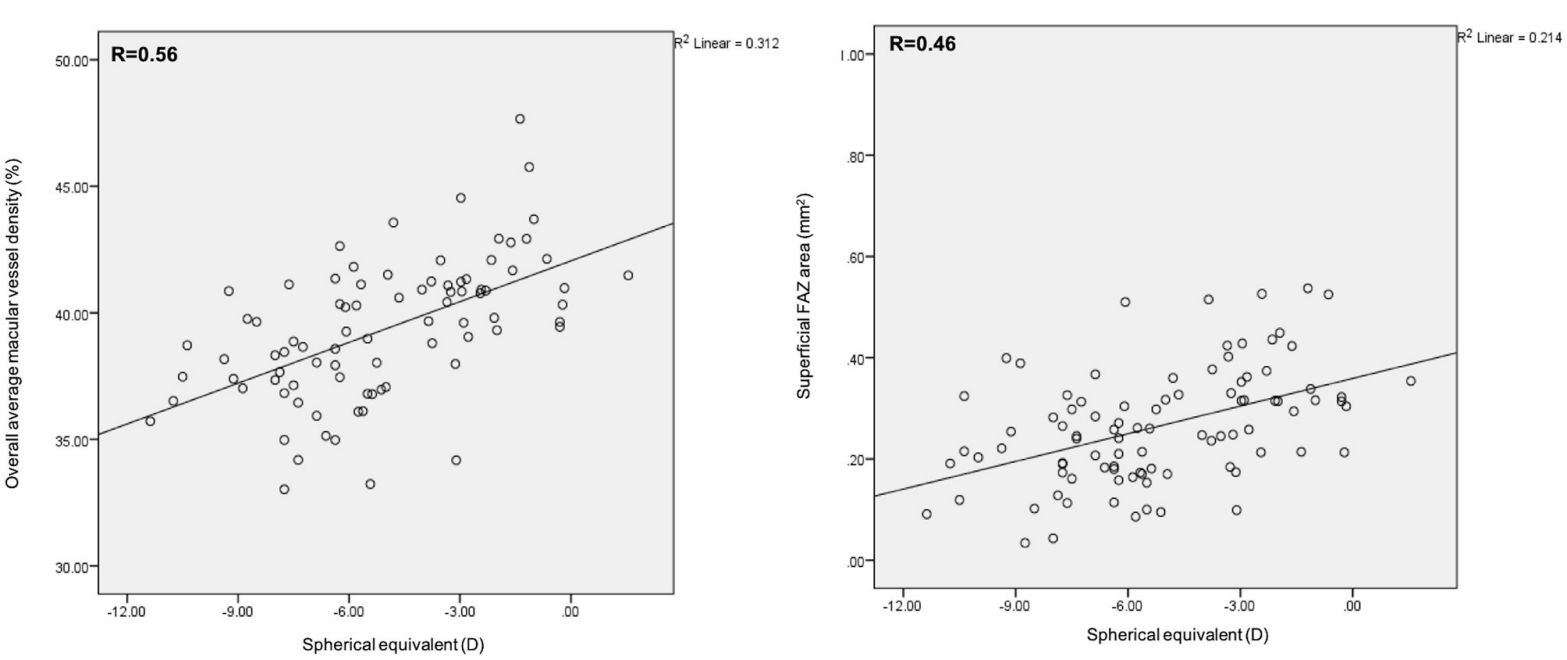
Scatterplots of overall vessel density and superficial FAZ area with SE.

Mean superficial FAZ area was 0.32 ± 0.11 mm^2^ and 0.22 ± 0.09 mm^2^ in the non-HM and HM groups, respectively. Mean deep FAZ area was 0.45 ± 0.13 mm^2^ and 0.48 ± 0.15 mm^2^ in the HM and non-HM groups, respectively. In ANCOVA analysis for the difference of the FAZ area between the two groups, the superficial FAZ area was smaller, but of borderline significance, in the HM group than in the non-HM group after adjusting for age and gender (*p* = 0.04) (Table 1). Table 2 shows that the difference in VD at various locations between the HM and the non-HM group. The mean overall macular VD was 40.9 ± 0.6% in non-HM group and 38.2 ± 0.5% in the HM group (*p* = 0.01 after adjusting for age and gender). There were no significant differences in the central, temporal, nasal, superior, and inferior VD after Bonferroni correction (Table 2). Both mean overall VD and superficial FAZ were positively correlated with more myopic SE (r = 0.56 and 0.46, respectively) (Figure 2).

**Table 1:** Difference of FAZ Area at different locations between high myopia and non-high myopia groups from both STARS study (*N* = 45) and SCORM study (*N* = 48).

	**All participants (*N* = 93) Mean μ (SD)**	**High myopia (*N* = 53)Mean μ (se)**	**Non-high myopia (*N* = 40) Mean μ (se)**	***P***
FAZ Area, mm^2^				
Superficial	0.27 (0.01)	0.22 (0.02)	0.32 (0.03)	0.04
Deep	0.47 (0.2)	0.45 (0.03)	0.48 (0.04)	0.70

*FAZ, Foveal avascular zone; SD, Standard deviation; se, standard error; μ, microns; mm, millimeter*.

*p-value: ANCOVA (adjusted for age and gender)*.

**Table 2:** Difference of vessel density at different locations between high myopia and non-high myopia groups from both STARS study (*N* = 43) and SCORM study (*N* = 42).

	**All participants (*N* = 85) Mean μ (SD)**	**High myopia (*N* = 47) Mean μ (se)**	**Non-high myopia (*N* = 38) Mean μ (se)**	***P***	
**Vessel density (%)**					
Overall average	39.3 (5.6)	38.2 (0.5)	40.9 (0.6)	0.01	
	**All participants (*****N*** **=** **85) Mean** **μ** **(SD)**	**High myopia (*****N*** **=** **47) Mean** **μ** **(se)**	**Non-high myopia (*****N*** **=** **38) Mean** **μ** **(se)**	**Bonferroni uncorrected** ***P***	**Bonferroni corrected** ***P***
**Vessel density (%)**					
Central 1 mm	20.7 (5.2)	18.8 (1.2)	23.2 (1.4)	0.07	0.42
Temporal	36.4 (7.7)	33.6 (1.8)	40.0 (2.1)	0.07	0.43
Nasal	40.6 (8.6)	39.6 (2.0)	41.8 (2.4)	0.57	1.00
Superior	49.8 (8.1)	47.3 (1.7)	53.4 (2.0)	0.07	0.44
Inferior	39.3 (9.8)	39.9 (2.1)	38.5 (2.5)	0.74	1.00

*SD, Standard deviation; se, standard error; μ, microns; mm, millimeter*.

*Uncorrected p-value: ANCOVA (adjusted for age and gender): Corrected p-value: Bonferroni correction for multiple comparisons of six measurements*.

## Discussion

In this study, we found decreased overall VD and smaller superficial FAZ area in high myopes compared with non-high myopes, independent of age and gender. Overall VD and superficial FAZ area correlated positively with more myopic SE. To our knowledge, this is one of the first studies utilizing SS-OCTA to measure macular VD in high myopes derived from population based cohorts.

To date, all of the OCTA studies of macular microvasculature in high myopes have been performed with SD-OCTA. One of our study objectives was to assess if the results of these studies could be validated using a SS-OCTA system. SS-OCTA is a relatively new development, with only two commercially available systems: PLEX Elite (PLEX Elite 9000, Version 1.6.0.21130; Carl Zeiss Meditec, Jena, Germany) based on optical microangiography (OMAG) and Triton (Topcon DRI OCT Triton Swept source OCT; Topcon, Tokyo, Japan) using the OCTA ratio analyses (OCTARA) algorithm. Both systems have demonstrated good reproducibility for macular VD measurements (28). Advantages of SS-OCTA imaging over SD-OCTA includes (29): (1) faster scan speeds allows for denser scans and larger scan areas compared with SD-OCT. A denser scan pattern will increase detection rate of flow signals within a given scan area. In addition, OCTA interprets flow as differences in signals between two consecutive OCT B scans separated in time, so a higher scan speed will facilitate detection flow signal in vessels where the flow rate is faster (30); (2) the longer wavelength and reduced sensitivity roll-off enhances detection of signals from the deeper retinal and choroidal layers; (3) the longer wavelength of SS-OCT is safer for the eye so a higher laser power can be used to penetrate the deeper vascular layers. In eyes with PM, SS-OCT was better than SD-OCT in visualizing outer retinal anatomy, and revealed abnormalities not seen on SD-OCT along the posterior staphyloma walls (3). SS-OCTA was also better than SD-OCTA for visualization of the extent of choroidal neovascular complexes (31). This study was preformed because the advantages of SS-OCT, particularly in highly myopic eyes, may potentially enhance detection of retinal microvascular structures. Further studies are needed to confirm this.

Previous studies of the retinal microvasculature in high myopes using OCTA have shown inconsistent results. These studies are summarized in Table 3. All of these studies were performed in hospital-based cohorts and were performed using 3 × 3 mm SD-OCTA scans. However, there is heterogeneity in terms of age, OCT instrument used and the methods by which VD was measured and calculated. Some studies included eyes with PM while others have excluded them. It is also unclear why high myopes were attending the eye clinic in these hospital based studies, and it is possible that some of these subjects had clinical pathology. Also, and most of these studies included more severe myopia than in our population based study (Table 3).

**Table 3:** A summary of studies of the macular microvasculature in high myopes using optical coherence tomographic angiography.

**Study**	**Sample size**	**Criteria**	**Age (years)**	**Refraction/axial length**	**Imaging modality**	**Retinal vascular parameter**	**Results**
Yang et al. (14)	33 HM 47 mild myopes/EM	HM: <-6D Controls: +0.5 to −3D MMD excluded	18–40	−8.68 ± 1.87D/27.11 ± 1.27 mm	Optovue (SSADA) 3 × 3 mm	Fractal analysis of superficial, deep and whole macular vascular plexi	VD was significantly less in all layers in HM. AL was negatively correlated with vascular density
Li et al. (15)	20 HM 20 controls	HM: <-5D Controls: >-3D MMD excluded	28 ± 5	−6.31 ± 1.23D/26.44 ± 0.97 mm	Angioplex (OMAG) 3 × 3 mm, Retinal function imager	Fractal analysis and blood flow velocity	VD but not blood flow velocity was lower in HM, AL was negatively correlated with vascular density
Fan et al. (17)	28 HM 33 MM 30 Controls	HM: ≤-6D MM: ≤-3D and >-6D, Controls: <3D and >-3D MMD not excluded	36.3 ± 14.7	−11.63 ± 5.36D/29.01 ± 2.69 mm	Optovue (SSADA) 3 × 3 mm	Macular VD	Highest VD in Controls followed by MM and lowest in HM. VD associated with both AL and SE
Venkatesh et al. (20)	86 (AL range 21.77–32.28 mm)	1.75D to −20D MMD exclusion unclear	10–44	−7.17 ± 5.71D/25.95 ± 2.41 mm	Optovue (SSADA) 3 × 3 mm	Macular VD	Negative correlation between VD and AL, positive correlation with SE and visual acuity
Yang et al. (21)	81 mild myopia 117 MM 70 HM	HM: ≤-6D MM: ≤-3D and >-6D Controls: <3D and >-3D	18–32	**–**7.14 ± 0.94D/26.15 ± 0.93 mm	Optovue (SSADA) 3 × 3 mm	Macular VD	No difference in superficial or deep macular VD
Mo et al. (19)	45 EM 41 HM 45 PM	EM: 0.50D to −0.50D HM: ≤-6D, without MMD PM: ≤-6D and AL ≥26.5 mm with MMD	38.3 ± 13.1	HM: −6.90 ± 1.23D/25.93 ± 0.58 PM: **–**15.22 ± 3.79D/29.55 ± 1.73 mm	Optovue (SSADA) 3 × 3 mm	Macular VD	Compared with the EM group, VD in the macular and arcuate fiber region was 1. not decreased in the HM group. 2. decreased in the PM group AL was negatively correlated with both superficial and deep macular VD
Milani et al. (18)	42 HM 40 controls	HM: SE ≥-6D Controls: 0 ± 2D MMD excluded	51.85 ± 10.87	– 10.26 ± 3.83D	Optovue (SSADA) 3 × 3 mm	FAZ area Macular VD Outer retinal flow area	HM had lower whole superficial VD and higher flow area in the outer retina No difference in FAZ area. SE was positively correlated with superficial VD and negatively correlated with outer retina perfusion
Al Sheikh et al. (16)	50 HM 34 Controls	HM: ≤-6D and AL ≥26.5 mm MMD excluded	25–83	−8.29 ± 2.94D	Optovue (SSADA) 3 × 3 mm	Macular VD and fractal analysis	VD and fractal dimension of the retinal capillary microvasculature were significantly lower in myopic eyes

*HM, high myopia; MM, moderate myopia; EM, emmetropia; PM, pathologic myopia; AL, axial length; D, diopter; SE, spherical equivalent; MMD, myopic macular degeneration; SSADA, split spectrum amplitude decorrelation angiography; OMAG, optical microangiography; FAZ, foveal avascular zone; VD, vessel density*.

Most of these studies (14–18, 20), including ours, have found decreased macular VD in high myopes than non-high myopes or emmetropes. The most plausible explanation for a reduction in macular VD is the stretching of the macular microvasculature in axially elongated eyes, leading to reduced VD rather than a loss of perfusion. This hypothesis is supported by Li et al.'s findings of reduced macular VD but preserved blood flow velocity in high myopes (15). They also found that decreased VD was significantly correlated with refractive error but not AL. Thus, refractive error itself may represent a different mechanism, unrelated to ocular stretching, by which VD was reduced. Other studies that analyzed fractal dimensions, a measure of the branching complexity of the microvasculature, found reduced branching of the macular vasculature in high myopes (14–16). This finding is consistent with the hypothesis that retinal vessels are stretched with increasing AL.

A few studies have found the converse to be true. Venkatesh et al. described a positive correlation between the VD and flow area index in both the superficial and deep vessel plexus with increasing AL and myopic refraction (20). They postulated that thickening of the inner retinal layers and outer plexiform layers in eyes with longer AL and high myopic spherical refraction observed in their study might have resulted in the higher VD and flow area indices in both the superficial and deep vessel plexus with more severe myopia. Similar results were reported by Mo et al. whereby macular flow density did not differ between high myopes and emmetropes, but was negatively correlated with AL (19). In addition, they observed a decreased macular flow density in eyes with PM compared with HM and emmetropia. Both studies differed from ours in the inclusion of older participants and the use of SD-OCTA rather than SS-OCTA. Lastly, Yang et al. demonstrated that refractive error did not affect the macular vascular density in myopic eyes (18–32 years) without pathologic changes (21).

We found a smaller FAZ area in the superficial vessel plexus in high myopes compared to non-high myopes, although the association was small and of borderline significance. Our findings contrasted with other reports in the literature. Li et al. measured the superficial FAZ area with the Zeiss HD-OCT with Angioplex™ OCTA device (Carl Zeiss Meditec, Dublin, CA) and found no significant difference between myopes and controls (0.28 ± 0.12 mm^2^ vs. 0.28 ± 0.13 mm^2^, *P* > 0.05) (15). The calculated FAZ diameter in the myopia group was 0.59 ± 0.12 mm compared to the control group (0.58 ± 0.12 mm, *P* > 0.05). In another study, Milani et al. found no difference in superficial FAZ area (0.23 ± 0.1 mm^2^ vs. 0.26 ± 0.1 mm^2^, *P* = 0.12) in high myopes compared to controls, and no correlation of FAZ area with spherical correction (18). In their study, FAZ area was measured automatically using the AngioVue, Angioanalytics, XR Avanti device (Software V.2016.1.0.26 Optovue Inc., Fremont, CA, USA). Although both groups obtained similar findings, Li et al. corrected for ocular magnification using Bennett's formula while Milani et al. did not. Axial length can result in measurement errors on confocal scanning laser ophthalmoscopy instruments, and may explain the difference in superficial FAZ area in our study (32).

Our study was limited by the cross-sectional design and a relatively small sample size. Two studies with similar refraction methods were combined to increase power but there may be heterogeneity between studies, i.e., the age difference between the two groups. Although we have adjusted for age in our analysis, this remains an important limitation of the present study. We did not assess the relationship between retinal microvascular parameters with visual acuity or retinal layer thickness measurements as these were beyond the scope and aims of this study. The strengths of our study includes the enrolment of participants from population based cohorts and the use of SS-OCTA to assess the retinal microvasculature.

## Conclusions

In conclusion, VD was lower and superficial FAZ area was smaller, in adolescents and young adults with HM compared to non-HM. Longitudinal studies in population based cohorts of high myopic individuals using different OCT-A imaging instruments may shed further light on the impact and validity of these findings on the subsequent development of PM.

## Data Availability Statement

The raw data supporting the conclusions of this article will be made available by the authors, without undue reservation.

## Ethics Statement

The studies involving human participants were reviewed and approved by SingHealth Centralized Institutional Review Board. The patients/participants provided their written informed consent to participate in this study.

## Author Contributions

C-WW, MA, and S-MS: conceptualization. SM, HH, ST, and CT: formal analysis. S-MS: funding acquisition. HH, CT, MA, Y-LW, RA, and S-MS: methodology. C-WW, SM, HH, Y-LW, CS, and S-MS: writing—original draft. C-WW, SM, CT, MA, Y-LW, RA, CS, and S-MS: writing—review and editing. All authors contributed to the article and approved the submitted version.

## Conflict of Interest

Y-LW—employee of Essilor International, Singapore. The remaining authors declare that the research was conducted in the absence of any commercial or financial relationships that could be construed as a potential conflict of interest.
